# Non-Negligible Role of Trace Elements in Influenza Virus Infection

**DOI:** 10.3390/metabo13020184

**Published:** 2023-01-26

**Authors:** Shan Xu, Duanyang Wang, Wenqi Zhao, Qinglin Wei, Yigang Tong

**Affiliations:** College of Life Science and Technology, Beijing University of Chemical Technology, Beijing 100089, China

**Keywords:** influenza virus, trace elements, antiviral treatments, selenium, immune responses, adjuvants

## Abstract

Influenza virus has continuously spread around the globe for more than 100 years since the first influenza epidemic in 1918. The rapid and unpredictable gene variation of the influenza virus could possibly bring about another pandemic in future, which might threaten to overwhelm us without adequate preparation. Consequently, it is extremely urgent to identify effective broad-spectrum antiviral treatments for a variety of influenza virus variants. As essential body components, trace elements are great potential candidates with an as yet poorly understood ability to protect the host from influenza infection. Herein, we have summarized the present state of knowledge concerning the function of trace elements in influenza virus replication along with an analysis of their potential molecular mechanisms. Modulation of host immune responses to the influenza virus is one of the most common modes to achieve the anti-influenza activity of trace elements, such as selenium and zinc. Simultaneously, some antioxidant and antiviral signal pathways can be altered with the participation of trace elements. More interestingly, some micro-elements including selenium, zinc, copper and manganese, directly target viral proteins and regulate their stability and activity to influence the life cycle of the influenza virus. Further verification of the antiviral effect and the mechanism will promote the application of trace elements as adjuvants in the clinic.

## 1. Introduction

**Figure 1:**
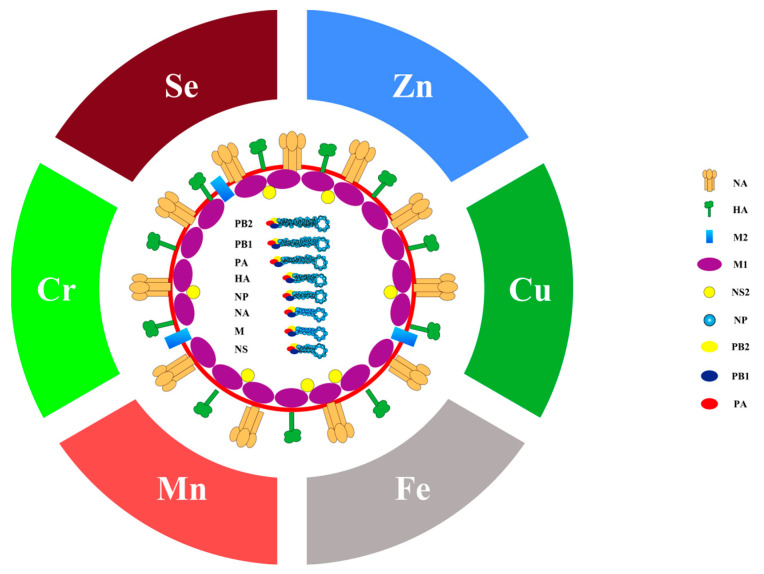
Diagram of the influenza A virus and its related trace elements. This figure represents the structure of influenza A virus. Hemagglutinin (HA) and neuraminidase (NA) on the membrane form spikes, matrix protein M2 embedded in the membrane as ion channels, matrix protein M1 is inside the virion to support the membrane, and non-structural protein 2 (NS2) bind to M1. The virus genome, in the form of ribonucleoprotein (RNP), is wrapped by nucleoprotein (NP), which relies on RNA polymerase complex (PB1, PB2 and PA) to replicate.

Seasonal influenza, caused by the influenza virus, leads to an enormous number of deaths every year around the world. It has been estimated that annual epidemics of seasonal influenza result in 3 to 5 million severe illnesses and 290,000 to 650,000 respiratory deaths [1]. The influenza virus is classified into four types, including type A, B, C and D. Influenza A virus is the most common type and historic pandemics were all caused by it [2]. Typically, influenza A is spherical, but filamentous in some cases [3]. As a member of the Orthomyxoviridae family, influenza A virus is enveloped by a lipid bilayer (Figure 1) [4]. The membrane is covered by hemagglutinin (HA) and neuraminidase (NA) to form the spikes. The matrix protein M1 forms an endoskeleton inside the lipid membrane to provide rigidity to the virion. The matrix protein M2 penetrates the envelope and forms a proton selective ion channel. The normal activity of the viral proteins guarantees the process of the influenza virus infection and replication.

Generally, there are two ways to combat influenza virus infection in clinics, including vaccination and drugs. Weakened or inactivated virus, as well as virus proteins, have been designed as antigens of a vaccine. Antiviral drugs can combat influenza viruses through interfering with the virus cycle by targeting virus protein activity or key infection processes. There are four FDA (Food and Drug Administration)-approved influenza antiviral drugs recommended by CDC (Centers for Disease Control) for combating recently circulating influenza viruses [5]. Those are Rapivab (peramivir), Relenza (zanamivir), Tamiflu (oseltamivir phosphate, also available as a generic drug), and Xofluza (baloxavir marboxil). The former three drugs target NA and Xofluza on endonuclease. However, due to the continuous mutation of viruses, new strains of influenza virus can be resistant to vaccines or drugs [6,7,8]. As a result, it would be very significant to find a broad-spectrum anti-influenza treatment.

Trace elements occupy less than a ten-thousandth of total body elements, but they are extremely indispensable for human life, including growth, development, and metabolism [9,10]. A deficiency of trace elements is closely related to various diseases [11,12,13,14]. For instance, selenium deficiency is associated with cardiovascular, thyroid and myodegenerative disease [15,16,17]. Specific roles of zinc were indicated in the pathological process of a variety of organs, such as liver and the gastrointestinal system, the cardiovascular and pulmonary systems, as well as the central and peripheral nervous systems [18,19,20]. Anemia is the most common symptom caused by iron deficiency [21]. Moreover, iron deficiency has also been identified as a vital therapeutic target for heart failure, with a reduced ejection fraction [22]. Significantly, although their essential role has been determined, an overdose of trace elements can be toxic to the human body. In most cases, trace elements are consumed from daily diets and are transported through the gastrointestinal tract before their entry into other organs [23]. Additionally, some commodities that directly contact the skin can be another source for absorption of trace elements [24]. As a result, the oral consumption of trace elements should be restricted to the amount recommended by dietary guidelines. Moreover, products with direct contact to skin, such as cosmetics, are supposed to meet strict criteria about the amount of trace elements.

In addition, trace elements are essential to maintain the normal immune responses to virus infection [25,26,27]. Multiple pieces of evidence indicate that some trace elements help the host to fight off the influenza virus by targeting the host or the virus proteins. As a potential broad-spectrum anti-influenza agent, trace elements should not be mostly ignored anymore. To fully investigate the anti-influenza function of trace elements, we have carried out an exploratory search using the Science Direct database (available at https://www.sciencedirect.com) (accessed on 1 October 2022) and PubMed database (available at https://pubmed.ncbi.nlm.nih.gov/) (accessed on 1 October 2022). The results include journal articles and book chapters. Additionally, Microsoft Bing was also used to search the related information on the website (available at https://cn.bing.com/) (accessed on 1 November 2022). The keywords “influenza” and “one of the trace elements” were used in the search box. On this basis, all related studies were obtained. We analyzed the titles and abstracts to filter for the studies that were relevant to the effects of trace elements on influenza virus. To make the review easier to understand, we classified the main text by six trace elements. At each stage, we first summarized their effects on the influenza virus, and then we analyzed the molecular mechanism of how they achieve their antiviral function. The mechanism consists of the host and viral targets, which will be discussed. This review stimulates the development of the combination therapy for influenza virus infection and provides novel ideas for the treatment of other viral infections.

## 2. Selenium-Deficiency Enhance the Replication of Influenza Virus

As a kind of essential trace element, selenium (Se) was revealed to play an important role in influenza infection. Se-deficiency could result in more serious apoptosis of the bronchial epithelial cells compared with Se-adequate cells after being infected with the influenza A/Bangkok/1/79 [28]. Selenium nanoparticles were proved to protect MDCK cells from apoptosis induced by H1N1 [29]. In addition, dietary selenium can help animals protect against the influenza virus. Selenium supplementation can protect chicken against avian influenza virus [30]. It was also shown that supplementary dietary selenium could protect mice from lethal influenza infection, and the survival rate was highly Se dose-dependent [31]. Similarly, Beck reported that Se-deficient mice presented serious pathology when infected with Influenza A/Bangkok/1/79, while relatively less inflammation was stimulated in the Se-adequate mice [32]. 

**Figure 2:**
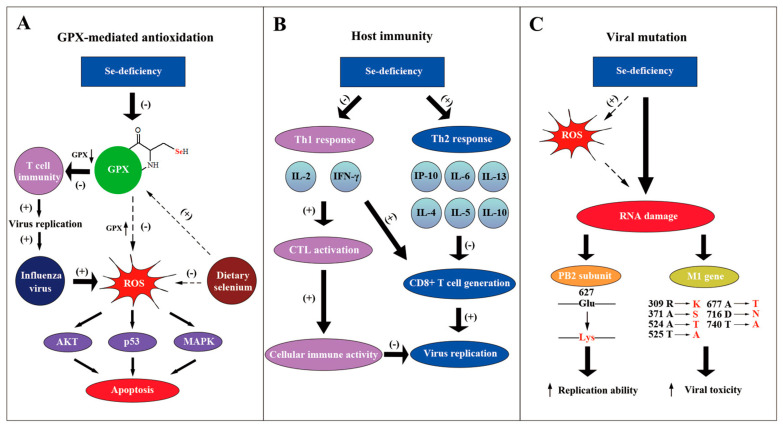
Molecular mechanisms of selenium against influenza virus. (**A**) The GPX-mediated antioxidation pathway participates in the anti-influenza process of selenium. Selenium functions as the vital part of GPX, which eliminates ROS. When selenium is deficient, T cell immunity is inhibited, which promotes the replication of the influenza virus. Moreover, selenium deficiency also leads to the apoptosis of the host cells through the AKT, p53 and MAPK pathways and aggravates viral cytotoxicity; supplementary selenium can suppress this negative effect. (**B**) Se-deficiency can cause the immune conversion from a Th1 response to a Th2 response. Cytokines such as IL-4, IL-5, IL-10 represented by the Th2 response whose mRNA level increased. This results in the suppression of CD8+ T cells and consequently promotes the influenza virus infection. Simultaneously, reduced Th1 responses suppress the cellular immune activity, which also increases the viral replication. (**C**) The influenza virus genome mutates when there is a lack of selenium. ROS caused by Se-deficiency, as one of the possible mechanisms, can directly damage the viral genome and give rise to mutations. It was demonstrated that PB2 gene and M1 gene could mutate to produce viral strains that had a stronger replication ability and a higher toxicity in a low selenium status.

One possible reason for selenium anti-influenza effects is that selenium forms the catalytic center of selenoenzymes (e.g. GPX1-6), which belong to antioxidase and can protect cells against reactive oxygen species (ROS) damage (Figure 2A) [33]. As the vital component of selenoenzymes, selenium deficiency dramatically decreased the activity of the GPX1 [28]. GPX activity in splenic cells is partly recovered through supplementary seleno-chemical sources [34]. Moreover, it was indicated that influenza virus can cause oxidative stress, which is harmful to the host gene [35]. Gong revealed that sodium selenite can inhabit the apoptosis induced by H1N1 through the possible pathways of AKT, p53 and MAPK, mediated by ROS [36]._._ Selenoproteins were demonstrated to mediate T cell immunity through the antioxidant mechanism [37]. T cell proliferation was suppressed when selenoproteins were deficient. The functions and mechanism of selenoproteins in influenza virus infection still need to be further investigated.

The effect of selenium on the host immune response also accounts for its anti-influenza function (Figure 2B). When selenium is deficient in mice, the level of interleukin 6 (IL-6) increases substantially, but interferon-induced protein 10 (IP-10) is down-regulated during the influenza infection [28]. Another research paper showed that Se-deficiency increased the mRNA level of cytokines like IL-4, IL-5, IL-10 and IL-13, whereas it decreased the level of IL-2 and interferon-γ (IFN-γ) in influenza-infected mice [32]. IL-2 and IFN-γ were reported to be necessary in activating cytotoxic T cells (CTL) to promote cellular immune activity [38]. Moreover, increased IL-4, 5, 10 were suggested to be a Th2 response, which suppresses CD8+ T cell generation [39]. Consequently, Se-deficiency decreases the immune response to the influenza virus with the participation of the host cytokines. On the contrary, selenium supplements promoted the production of the tumor necrosis factor α (TNF-α) and interleukin-1-beta (IL1-β) by macrophages [40]. The increased proinflammatory cytokines may play an important role in anti-influenza processes. In conclusion, the alteration of cytokines by selenium concentration influences the host inflammatory response to the influenza virus.

Apart from the effect on host immune reaction, the influenza virus can mutate under the condition of Se-deficiency (Figure 2C). E627K mutation at PB2 subunit of the influenza virus, which strengthens the replication ability of polymerase complex, was discovered to be related to host Se-deficiency [41,42,43]. Pathogenic avian influenza H5N1 virus with K627 in PB2 was found in regions of poor Se bioavailability, such as the Qinghai and Hubei provinces of China, indicating the possibility of selenium-forced mutation in the influenza virus [44,45,46,47]. Moreover, it was demonstrated that influenza virus mutation was forced by host selenium status [48]. Through gene sequencing, 29 nucleotide mutations were found in the M1 gene of the influenza virus derived from Se-deficient mice, possibly accelerating the viral growth cycle and enhancing the viral toxicity. Increased oxidative stress caused by selenium deficiency that directly damages viral RNA may be one possible mechanism. More effort should be made to further clarify the molecular mechanism of viral mutation, which may help to design methods to avoid a more infectious and toxic influenza genome [49]. 

## 3. Zinc Show Potential Anti-Influenza Activity by Decreasing HA Stability

It has been widely believed that zinc participates in various physiological and pathological processes, including virus infection [10,50]. Studies have shown that zinc has the potential to inhibit influenza virus infections by multiple possible mechanisms [51,52]. After influenza H1N1 infected MDCK-SIAT1 cells, the addition of zinc oxide nanoparticles (ZnO-NPs) significantly decreased the viral titer in the cell. However, the anti-influenza effect disappeared when ZnO-NPs were pre-exposed or co-exposed with the virus to the cell [53]. In addition to the in vitro experiments, studies have indicated good in vivo anti-influenza activity of zinc. With the treatment of ZnAL42, the survival rate of mice was obviously higher than untreated control mice after being infected with influenza H5N1 or H1N1 [54]. Moreover, a combination therapy of zinc and trimethoprim reduced the lethal effect of the influenza virus in chicken embryos. With a higher concentration, the combined compounds inhibit the binding affinity of viruses to the host cell receptor, which was reflected by the hemagglutination-inhibition (HAI) assay using chicken red cells [55]. Zinc also presented a positive reaction to swine influenza viruses (SIV). It was shown that high doses of zinc stimulated the humoral immune response of pigs after vaccination and help them to recovery from SIV infection [56]. More interestingly, zinc-embedded polyamide 6.6 (PA66) fibers obviously inactivated influenza H1N1 when the virus was absorbed in the materials [57]. These results provide more direct evidence to the anti-influenza effect of zinc and give a novel insight to develop a useful face mask to defend against the influenza virus. 

Some efforts have been made by researchers to study the mechanism of anti-influenza activity of zinc. Both the host and the influenza virus could be the target of zinc. It is well known that zinc is an essential element for human immune system, and plays a key role in the resistance to viral infections [26,50,58]. Zinc has been demonstrated to influence the development and activity of various immune cells [59]. For instance, zinc supplementation increased the serum zinc concentration in the elderly, enhancing the function of T cells [60]. Preincubation with zinc obviously improved the killing activity of the natural killer (NK) cells and zinc deficiency decreased the killing activity [61]. Zinc supplements can also induce the regulatory T cells (Treg) and decreased the production of IFNγ [62]. 

In addition, a variety of zinc-finger-containing proteins act as broad-spectrum anti-viral molecules, such as zinc finger antiviral protein (ZAP) and Zn-dependent metallopeptidase STE24 (ZMPSTE24) [26]. On the contrary, some zinc-containing proteins enhance the replication of viruses. As a zinc-finger-containing transcription factor, nuclear matrix protein 4 (NMP4) has been shown to regulate the antiviral immune responses to influenza virus [63]. *Nmp4* knockout significantly protected mice from influenza H1N1, decreasing monocytes and neutrophil infiltration. Similarly, zinc finger-containing cellular transcription corepressor (ZBTB25) promoted influenza A virus replication through stimulating the activity of the viral RNA-dependent RNA polymerase (RdRp) protein [64]. Zinc content might interfere with influenza virus replication via the zinc-containing proteins. Moreover, research showed that zinc reversed the apoptosis of Hela cells induced by the influenza virus, which may be another explanation for zinc antiviral functions [65]. 

**Figure 3:**
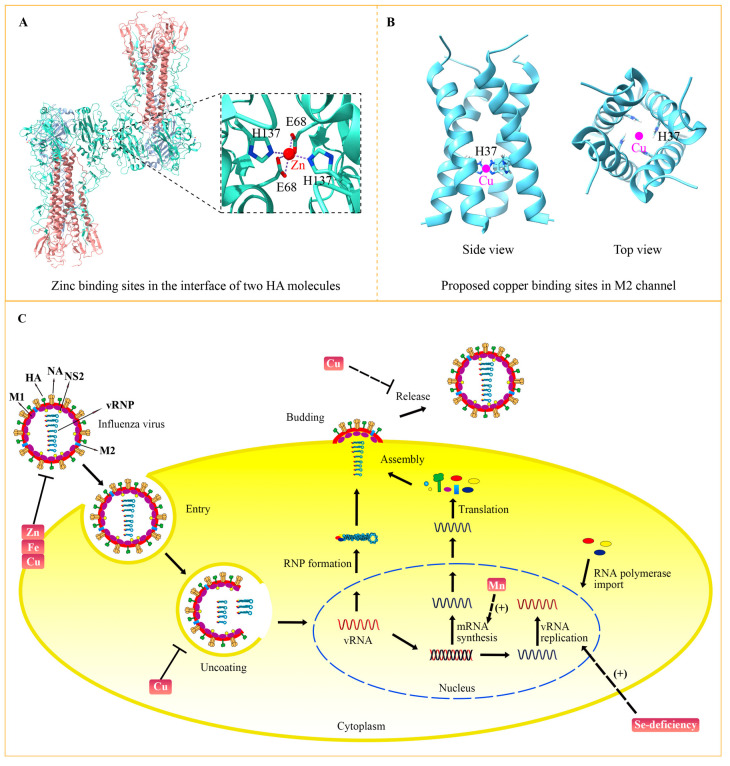
The targets of trace elements for the influenza virus. (**A**) Zinc resides in the interface of the HA proteins with the participation of two Glu68 and two His137 (PDB: 6LKS) [66]. A zinc ion is indicated with a red circle. (**B**) Copper is predicted to bind to His37 at the transmembrane domains of the influenza M2 channel. The proposed model is based on the NMR structure of M2 (PDB: 2KQT) [68]. The pink circles indicate copper ions that are predicted to reside in the center of the channel. (**C**) Influenza virus life circle and the targets of trace elements. The influenza virus enters the cell by endocytosis. The acidification in the endosomes promotes the uncoating process and releases viral RNPs into the nucleus. The viral gene was transcribed and replicated to produce mRNA and new viral RNA (vRNA) by the RNA polymerase complex. Then, viral mRNA is transported to the cytoplasm and translated into viral proteins, which are assembled into new viral RNPs with vRNA. The newly assembled virus is released from the host cells at the budding sites after sialic acid residues are cleaved by NA. Functions and targets of trace elements in viral life cycle are indicated.

In addition to its effects on the host, zinc status changes the virulence of influenza virus by targeting viral proteins. The crystal structure of the influenza virus hemagglutinin (HA) has revealed that zinc binds to Glu68 and His137 residues of HA (Figure 3A) [66]. Binding of high concentration zinc results in multimerization of HA and decreases its acid stability. Because conformational changes of HA are vital for influenza virus membrane fusion and replication, zinc-involved HA modulation might be one way to perform the anti-viral function of zinc. Additionally, it was indicated that zinc could bind to the M1 protein of the influenza A virus, which may play significant role in M1 activity and the virus-uncoating process [67]. The structural role of zinc in other influenza virus proteins need to be further studied, which would help to clarify the precise mechanism.

## 4. Copper Inactivate Influenza Virus by Targeting Key Viral Proteins

Apart from vaccines and drugs, the host metabolism has been targeted by some research into how to suppress the replication of influenza virus [69]. A variety of studies have indicated that copper-involved pathways in the host were effected after influenza virus infection [69,70]. Moreover, host cell copper transporters CTR1 and ATP7A have been demonstrated to be important for influenza virus replication, suggesting the role of copper in the influenza virus life cycle [71]. Indeed, more and more studies have revealed the anti-influenza effect of copper by targeting the host and virus [72]. As early as 1998, copper chelates were discovered to inhibit influenza virus-induced apoptosis in MDCK cells and restrain the release of the virus [73]. A group of scientists found two copper complex Cu(NO_3_)_2_*3H_2_O and Cu(NO_3_)_2_*H_2_O polymers had the potential to combat influenza A virus in macrophages [74]. With the treatment of these two compounds, the expression of interferon-inducible transmembrane proteins (IFITM) was induced. However, how IFITM interferes with influenza virus amplification was not discussed. 

In addition, copper could inhibit the influenza virus directly by affecting the activity of key viral proteins. Before identifying the molecular mechanism, multiple pieces of evidence had determined the direct anti-influenza effect of copper, which can inactivate the influenza virus when they were incubated together in vitro [75,76]. M2 is an essential membrane protein in the influenza virus, functioning as a proton-selective ion channel. Cu^2+^ could inhibit the proton-transport function of M2 by binding to the key active site His37 (Figure 3B) [77,78]. Chemical studies have shown the binding of copper to His37 disturbs histidine–water exchange, inhibiting the structural dynamics of histidine and eventually destroying the function of the M2 proton channel [79]. In addition to M2, HA and NA proteins that reside in the membrane of the influenza virus can also be inactivated by copper. For instance, the solid state of cuprous oxide (Cu_2_O) reduced the activity of HA and NA, thus inactivating the influenza virus [80]. With the development of nanodevices, a group of scientists synthesized copper nanoparticles (Cu Nps) and copper microparticles (Cu Mps) and evaluated their antiviral activities. Their results showed that both Cu Nps and Cu Mps can inactivate and degrade the influenza A virus, and Cu Nps was more effective than antiviral agents [81]. Copper can also be combined with other elements to obtain greater antiviral activity. It was demonstrated that copper-graphene (Cu-Gr) nanocomposites can clearly decrease the titer of influenza virus and its antiviral effect to a greater extent than Cu nanoparticles or graphene individually [82]. The hybrid possibly compromises the integrity of influenza virion particles, thus interfering with their attachment and entry into the host cells. 

## 5. Iron Inactivate Influenza Virus by Inducing Viral Lipid Peroxidation

In 1990, it was demonstrated that iron deficiency reduced antibody activity against the influenza virus in rats, most likely by causing defects in T helpers or T suppressors and/or in lymphokines connecting the T and B lymphocytes [83]. However, due to the limited condition, there had not been any conclusions drawn about humans at that time. Until, in 2020, a group of researchers first reported the link between disease severity and iron status of patients after an avian influenza virus H7N9 infection [84]. The study showed serum–iron level in H7N9-infected patients was obviously lower than that in healthy controls. In addition, after one or two weeks of post-infection, serum–iron concentration in patients who died later was much lower than patients who survived. It was the first indication from clinical data about the vital role of iron in combating the influenza virus in humans.

A variety of iron oxide nanoparticles (nanozymes) have been designed to be a novel dosage form of iron to investigate its anti-influenza effect and the molecular mechanism. Early in the 1970s, studies confirmed that iron oxide can effectively purify and concentrate Influenza A and B, indicating the high binding affinity between iron oxide and the influenza virus [85]. Because iron oxide did not always show a clinical benefit, nanotechnology has been applied to convert iron oxide into iron oxide nanoparticles. With the synthesized iron oxide nanoparticles ( IO NPs), researchers have proved their high antiviral response to influenza virus H1N1 [86]. With the treatment of IO NPs, H1N1 replication was significantly inhibited in MA104 cells through the detection of cell viability, plaque formation and viral transcripts. Additionally, IO NPs have also been applied as good vaccine adjuvants. A combination of carboxymethyl chitosan bound in iron oxide nanoparticles (CMC-IO NPs) with influenza H9N2 vaccine has been shown to enhance the immune response of mice [87]. The vaccinated mice showed high HA antibody titers and increased lympho-proliferative activity of re-stimulated splenic lymphocytes, as well as increased the concentration of IFNγ and IL2. Due to its enzyme-like property, IO NPs can also be called iron oxide nanozymes (IONzymes). It was demonstrated that IONzymes possess a broad-spectrum antiviral activity on many types of influenza A virus [88]. Mechanistic studies show IONzymes induced the lipid peroxidation of the virus envelope membrane and destroyed the integrity of HA, NA, and M1, which ultimately inactivated the influenza virus. More research is still required to further validate the anti-influenza effect of iron and the inside mechanism to demonstrate its clinical application.

## 6. Manganese Modulate Influenza Virus PA Activity

As an essential trace element, manganese participates in multiple biological processes. In recent years, more and more studies have demonstrated the indispensable role of manganese in immune responses to DNA virus infection [89,90]. In fact, manganese has the potential to defend against the RNA influenza virus. Early in 1996, it was proved that manganese superoxide dismutase (MnSOD) can suppress influenza A virus infections in mice [91]. However, the antiviral effect of MnSOD was virus dose-dependent. With a high viral challenge, the inhibition of the mice mortality rate by MnSOD became weak. Moreover, it was demonstrated that manganese salt (Mn jelly, MnJ) supplements could increase the protective effect of the influenza vaccine, reducing the mortality rate of mice when they were subsequently infected by H1N1 [90].

Whether manganese possesses a direct anti-influenza virus effect is still unclear, thereby hindering the process of clarifying its antiviral mechanism. Manganese exposure in the juvenile period activates the glial inflammatory responses to influenza virus H1N1 in mice [92]. Epigenetic alterations in glia may contribute to the inflammatory activation and the antiviral function of manganese. In addition, X-ray structure showed that influenza endonuclease inhibitor flavonoids bind to PA with the coordination of two manganese ions, indicating the possible role of manganese in maintaining PA endonuclease activity [92]. Indeed, the structural information of PA has demonstrated a direct interaction between PA and manganese [93,94]. Moreover, manganese could increase the thermal stability and endonuclease activity of PA, which indicates a positive effect of manganese for influenza virus replication [95]. Because manganese stimulated innate immunity and enhanced the cGAS-STING pathway to combat DNA virus [89], it was thought likely that manganese modulated the host immune responses to the influenza virus. All-in-all, whether and how manganese influences influenza infection should be further studied.

## 7. Chromium Protect against Avian Influenza Virus

In 2014, the European Food Safety Authority (EFSA) declared that there is no evidence to show the beneficial effect of chromium (Cr) for health, and suggested Cr is not an essential trace element for humans [96]. In general, however, dietary Cr is believed to be helpful for some diseases, such as diabetes and hyperlipidemia [97,98]. Moreover, some research has indicated that Cr helps the host against the avian influenza virus. Antibody titers against avian influenza (AI) in 21- to 42-day old broiler chicken with additional chromium were higher than those with a control diet under heat stress [99]. Similarly, Lu et al. showed that broiler chicken with Cr supplements presented a stronger immune response to AI vaccines [100]. Those with dietary Cr gained more weight in the thymus and spleen and possessed a higher serum antibody titer. 

The anti-influenza immune activity of Cr may be mediated by the increased IL-2 that stimulated the lymphocyte transformation and improved the T lymphocyte percentage [100]. In addition, it was proved that supplementary Cr was associated with an inflammatory reaction through the alteration of TNF-α, IL-6 and C-reactive proteins (CRP) [101,102,103]. However, whether and how the inflammatory effect of Cr contributes to its anti-influenza activity is still lacking. Besides the immune and inflammatory effects, Cr can also serve as an important antioxidant under environmental stress [98,104]. It was documented that dietary Cr reduced the hepatic nuclear factor kappa-B (NF-κB), increasing the inhibitor of NF-κB α (IκBα) and heat shock protein 72 (HSP72), which alleviated the oxidative stress [105,106]. It is likely that diary Cr potentially helps to eliminate the ROS caused by influenza virus; this needs to be verified in the future.

## 8. Discussion

The life cycle of the influenza virus involves multiple processes, including entry, uncoating, viral gene replication, assembly, budding and release (Figure 3C) [4]. Inhibition of each process can suppress virus multiplication. Zinc, copper and iron can inactivate the influenza virus by impairing viral proteins. A high concentration of zinc leads to multimerization of HA and disrupts its stability. Copper has been demonstrated to decrease the activity of HA and NA, consequently inactivating the influenza virus. As a kind of oxidizing agent, iron causes the lipid peroxidation of the viral membrane, which destroys the integrity of HA, NA and M1. After entering the host cell by endocytosis, the influenza virus is trafficked to the lysosome. The acid environment of lysosome activates the M2 channel, thereby inducing the fusion between lysosome and viral membrane, which releases the viral genome into the nucleus to accomplish viral gene replication. Copper could inhibit the transport function of M2, probably by binding to the key active site His37, which suppresses the uncoating and fusion process. Transcription and replication of viral genes rely on the polymerase complex, including PB1, PB2 and PA. Selenium-deficiency is closely related to the mutation of PB2 and M1, which increases virulence and viral replication ability. Manganese directly binds to PA and helps to stabilize its endonuclease activity. Because of the essential role of PA in viral gene transcription, manganese tends to be indispensable to the synthesis of viral mRNA. After the budding of assembled viral progeny, the virus is released with the help of NA to disrupt the interaction between virus and host membrane receptors. Considering that copper can decrease the activity of NA, copper may play a role in the release of progeny virions. A combination therapy of diverse trace elements with different viral targets may promote their anti-influenza efficiency.

In addition to the trace elements mentioned above, other trace elements like magnesium and iodine may also help to resist influenza virus infection, but more evidence is required [107,108,109,110,111]. The crystal structure of the N-terminal of the PA subunit has revealed a direct interaction between the PA and magnesium ions [107]. Studies have shown that manganese has a much stronger affinity to PA than magnesium ions, which weakens the role of magnesium in regulating PA activity [108]. However, considering that the concentration of magnesium is much higher than that of manganese under physiological conditions, magnesium ions may co-mediate the activation of PA endonuclease. In addition, it is indicated that interaction between the N terminal domain of PA and substrate RNA depends on the presence of Mg^2+^ [109]. Whether magnesium modulates PA activity or interferes with the replication of the influenza virus needs to be validated by functional experiments. As an effective broad-spectrum antiseptic, iodine has been used to kill bacteria, viruses, and fungi for a long time [110]. Probably because high-doses of iodine taken orally is harmful for human body, iodine has mostly been studied in vitro. The anti-influenza function of iodine in vivo remains to be studied. With more data being obtained about the anti-influenza efficiency of various trace elements, there is a likelihood that combinations of several trace elements may achieve a much better antiviral effect. From this perspective, some medicinal plants and herbal medicines that are rich in many trace elements may have the potential to combat the influenza virus [111].

## 9. Conclusions

In this review, we have summarized the anti-influenza virus roles of essential trace elements, including selenium, zinc, copper, iron, manganese and chromium. The elements that directly target and inactivate the influenza virus could be used to design protective equipment such as face masks that specifically protect against the influenza virus. Furthermore, some trace elements possess broad-spectrum antiviral capabilities by stimulating the host immune activities or other signal pathways. It is worth investigating their antiviral functions in other virus, especially for the newly emerging virus infections that lack effective treatments in clinic. For the field of chemistry, this work provides new ideas for the synthesis of chemicals with anti-influenza functions. Specific metal chelation in the chemical structures may obviously enhance their antiviral activity. Simultaneously, this review has further demonstrated that trace elements could be developed as adjuvants in vaccines and drugs to promote their antiviral effect in clinic. In conclusion, we should pay more attention to the anti-influenza function of trace elements. Further validation of their antiviral efficiency and determination of the molecular mechanism will promote the application of trace elements in preventing virus infections and prepare us for the potential influenza epidemics of the future.

## Abbreviations

HA: hemagglutinin, NA: neuraminidase, NS2: non-structural protein 2, NP: nucleoprotein, RNP: ribonucleoprotein, FDA: Food and Drug Administration, CDC: Centers for Disease Control, ROS: reactive oxygen species, IL-6: interleukin 6, IP-10: interferon-induced protein 10, IFN-γ: interferon-γ, CTL: cytotoxic T cells, TNF-α: tumor necrosis factor α, IL1-β: interleukin-1-beta, ZnO-NPs: zinc oxide nanoparticles, HAI: hemagglutination inhibition, SIV: swine influenza viruses, PA66: polyamide 6.6, NK: natural killer, Treg: regulatory T cells, ZAP: zinc finger antiviral protein, ZMPSTE24: Zn-dependent metallopeptidase STE24, NMP4: nuclear matrix protein 4, RdRp: RNA-dependent RNA polymerase, IFITM: interferon inducible transmembrane proteins, IONzymes: iron oxide nanozymes, MnSOD: manganese superoxide dismutase, EFSA: European Food Safety Authority, AI: avian influenza, CRP: C-reactive protein, IκBα: inhibitor of NF-κB α, HSP72: heat shock protein 72, NF-κB: nuclear factor kappa-B.
